# *In situ* SERS reveals the route regulation mechanism mediated by bimetallic alloy nanocatalysts for the catalytic hydrogenation reaction†

**DOI:** 10.1039/d2sc06808g

**Published:** 2023-02-27

**Authors:** Xiaoxiao Li, Jinghua An, Ze Gao, Chang Xu, Yaoying Cheng, Simin Li, Lu Li, Bo Tang

**Affiliations:** a College of Chemistry, Chemical Engineering and Materials Science, Collaborative Innovation Center of Functionalized Probes for Chemical Imaging, Key Laboratory of Molecular and Nano Probes, Ministry of Education, Shandong Normal University Jinan 250014 P. R. China tangb@sdnu.edu.cn lilu5252@163.com

## Abstract

Synthesizing arylamines with high selectivity *via* hydrogenation of nitroaromatics is a long-standing challenge because of the complex reaction pathways. Revealing the route regulation mechanism is the key to obtain high selectivity of arylamines. However, the underlying reaction mechanism of route regulation is uncertain owing to a lack of direct *in situ* spectral evidence of the dynamic transformation of intermediate species during the reaction process. In this work, by using *in situ* surface-enhanced Raman spectroscopy (SERS), we have employed 13 nm Au_100−*x*_Cu_*x*_ nanoparticles (NPs) deposited on a SERS-active 120 nm Au core to detect and track the dynamic transformation of intermediate species of hydrogenation of *para*-nitrothiophenol (*p*-NTP) into *para*-aminthiophenol (*p*-ATP). Direct spectroscopic evidence demonstrates that Au_100_ NPs exhibited a coupling route with the *in situ* detection of the Raman signal assigned to coupling product *p*,*p*′-dimercaptoazobenzene (*p*,*p*′-DMAB). However, Au_67_Cu_33_ NPs displayed a direct route without the detection of *p*,*p*′-DMAB. The combination of X-ray photoelectron spectroscopy (XPS) and density functional theory (DFT) calculations reveals that Cu doping can favor the formation of active Cu–H species owing to the electron transfer from Au to Cu, which can promote the formation of phenylhydroxylamine (PhNHOH*) and favor the occurrence of the direct route on Au_67_Cu_33_ NPs. Our study provides direct spectral evidence demonstrating the critical role of Cu in route regulation for the nitroaromatic hydrogenation reaction at a molecular level and clarifies the route regulation mechanism. The results have significant implications for revealing multimetallic alloy nanocatalyst mediated reaction mechanisms and help to guide the rational design of multimetallic alloy catalysts for catalytic hydrogenation reactions.

## Abstract 2

*In situ* SERS provides direct spectral evidence of route regulation mediated by Au_100−*x*_Cu*_x_* NPs. Further XPS and DFT calculations reveals that Cu doping promotes Cu–H formation and electron transfer, avoiding the formation of intermediate *p*,*p*′-DMAB.

## Graphical abstract

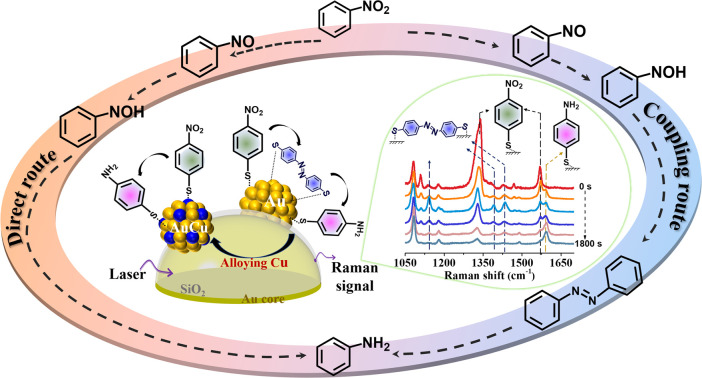

## Introduction

**Scheme 1:**
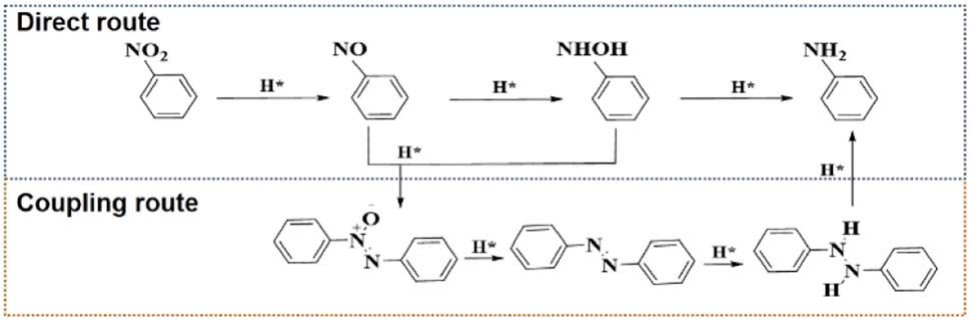
The complex reaction route for the reduction of a nitroaromatic compound to the corresponding aniline.

The hydrogenation reactions of nitroaromatic compounds is a typical reaction to prepare arylamines, which are very important industrial intermediates for dyes, medicines agricultural chemicals, and additives.^1^ However, synthesizing arylamines with high selectivity *via* hydrogenation reactions is a long-standing challenge because of the complex reaction pathways (Scheme 1).^2,3^ In the nitroaromatic hydrogenation process, arylamines can be synthesized by the direct route of nitroaromatic → nitrosoaromatic → arylhydroxylamine → arylamines. These metastable reaction intermediates will further undergo the condensation coupling route to form coupling derivatives such as arylazo compounds, which can lower the selectivity of arylamines. Therefore, designing catalysts for highly selective preparation of arylamines and revealing the route regulation mechanism is the key to obtain high selectivity of arylamines.

Gold (Au), which acts as a typical nanocatalyst, has received wide attention and has been regarded as an excellent hydrogenation catalyst owing to its good stability.^4^ However, the mild catalytic hydrogenation properties of Au nanoparticles (NPs) limit their further applications.^5^ In this regard, bimetallic alloy NPs have recently been prepared to improve the catalytic hydrogenation performance^6–10^ by route regulation.^11–13^ For example, the reaction pathway of hydrogenation reactions of nitroaromatic compounds can be altered by alloying copper into Au NPs under visible-light irradiation.^8^ However, the underlying reaction mechanism of route regulation is uncertain owing to a lack of direct *in situ* spectral evidence of the dynamic transformation of intermediate species during the reaction process. Although many techniques are available to study the reaction mechanism, the dynamic transformation of intermediate species during the reaction process is difficult to be tracked in real-time owing to their small adsorption amount and short lifetime.^12,14–17^ Therefore, a technique with high sensitivity, is needed urgently to *in situ* study the route regulation mechanism in real-time over bimetallic alloy NPs.

Surface-enhanced Raman spectroscopy (SERS) is a promising tool for *in situ* detecting surface species owing to its ultrahigh surface single-molecule sensitivity.^18–24^ During SERS measurement, the Raman signal from surface species can be amplified by using SERS-active Au^23,25^ or Ag.^26,27^ By using its high sensitivity, *in situ* SERS has been used to detect active species in biological systems by research groups including our group.^28–35^ Recently, it has begun to emerge in the study of the underlying catalytic reaction mechanisms.^36–38^ For example, by using bifunctional Au@Ni_3_FeO_*x*_ structures, the Raman signal from intermediate species (O–O^−^) during the oxygen evolution reaction on Ni_3_FeO_*x*_ NPs can be enhanced and seen because of the SERS enhancement from the Au core.^39^ Besides, the direct spectral evidence of active intermediate species (*OH and *OOH) in the oxygen reduction reaction was obtained on a metal Pt catalyst by using a Au core as the Raman signal enhancer.^40^ In addition, with the help of *in situ* SERS, the hydrogen spillover effect,^41^ size effect,^42,43^ as well as crystal effect^44^ were revealed by using the hydrogenation of *para*-nitrothiophenol (*p*-NTP) to *para*-aminthiophenol (*p*-ATP) as a model reaction. That said, SERS is a highly surface-sensitive technique and can be employed to *in situ* track the dynamic transformation of intermediate species on bimetallic alloy NPs.

Herein, with hydrogenation of *p*-NTP to *p*-ATP as the model reaction, we used *in situ* SERS to reveal the route regulation mechanism mediated by Au_100−*x*_Cu_*x*_ alloy nanocatalysts. Specifically, 13 nm single-component Au_100_ NPs, as well as bimetallic Au_90_Cu_10_ NPs and Au_67_Cu_33_ NPs were prepared and deposited on a SERS-active 120 nm Au core covered with a very thin layer of SiO_2_, forming Au@SiO_2_@Au_100_, Au@SiO_2_@Au_90_Cu_10_, and Au@SiO_2_@Au_67_Cu_33_, respectively. Here, the SiO_2_ shell is essential to prevent the interaction between the 120 nm Au core and analytical targets. Then, the Raman signal of surface intermediate species during hydrogenation of *p*-NTP to *p*-ATP on nanocatalysts can be amplified and detected due to the SERS enhancement properties of the 120 nm Au core. Moreover, we studied the changes in the Raman signal of intermediate species by varying the Cu content. As a result, the direct spectral evidence demonstrating that the reaction route of *p*-NTP to *p*-ATP regulated from the coupling route on Au_100_ NPs to the direct route on bimetallic Au_67_Cu_33_ NPs, was presented. By combining the *in situ* spectral evidence, X-ray photoelectron spectroscopy (XPS), and density functional theory (DFT) calculations, the underlying route regulation mechanism mediated by bimetallic Au_67_Cu_33_ NPs for the nitroaromatic hydrogenation reaction was further clarified.

## Results and discussion

**Fig. 1:**
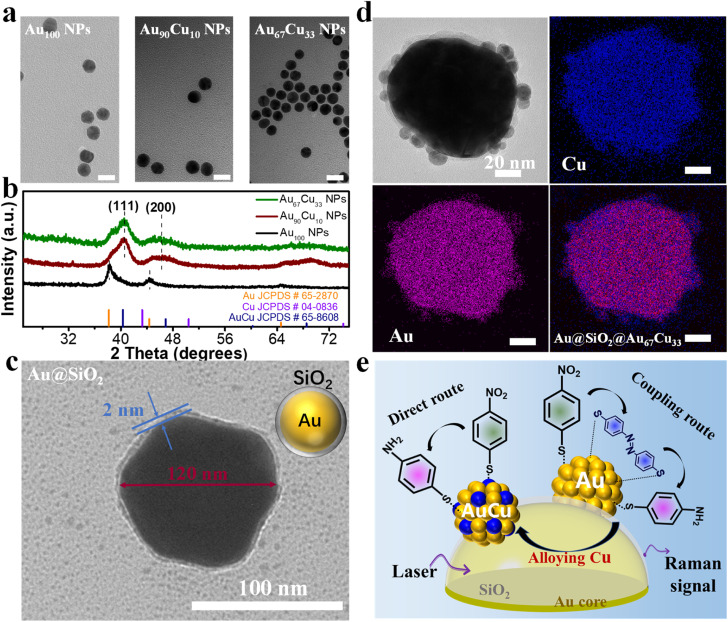
TEM images (a) and XRD patterns (b) of Au_100_ NPs, Au_90_Cu_10_ NPs and Au_67_Cu_33_ NPs. The scale bar in (a) is 20 nm. (c) TEM image of Au@SiO_2_. (d) TEM image and element mapping images of a single Au@SiO_2_@Au_67_Cu_33_. (e) Illustration of *in situ* SERS detection of the *p*-NTP hydrogenation process over Au@SiO_2_@Au or Au@SiO_2_@AuCu catalysts.

At the beginning, three nanocatalysts, including one single-component Au_100_ NPs and two bimetallic Au_100−*x*_Cu_*x*_ alloy NPs with different molar ratios of the Au atom and Cu atom, were prepared according to the literature report.^52^ Inductively coupled plasma mass spectrometry (ICP-MS) results showed that the molar ratios of Au and Cu for the two bimetallic Au_100−*x*_Cu_*x*_ NPs are 90 : 10 and 67 : 33, respectively (Table S2†). Thus, Au_100_ NPs, Au_90_Cu_10_ NPs, and Au_67_Cu_33_ NPs were successfully obtained. Transmission electron microscopy (TEM) characterization studies further showed that Au_100_ NPs, Au_90_Cu_10_ NPs, and Au_67_Cu_33_ NPs possessed almost identical shape and similar size (13 nm) (Fig. 1a and S1a−c†).^8^ X-ray diffraction (XRD) data of AuCu alloys with different compositions show the typical face-centered cubic crystal phase with a similar diffraction pattern between the standard Au and Cu peaks (Fig. 1b), which is consistent with the reported AuCu alloys in the literature.^52^ The high-resolution transmission electron microscopy (HRTEM) image of Au_67_Cu_33_ and Au_90_Cu_10_ shows an interplanar distance of about 0.22 nm, which matches well with the *d*-spacing value of the metallic AuCu(111) plane (Fig. S2†). Further study of the *p*-NTP hydrogenation process on Au_100_ NPs, Au_90_Cu_10_ NPs, and Au_67_Cu_33_ NPs using *in situ* SERS is limited due to their less-SERS enhancement properties owing to their small size (Fig. S3†).^45^ In this regard, SERS-active Au NPs with a diameter of 120 nm were prepared as the SERS enhancer (Fig. S4a and b†).^45,46^ After that, the Au@SiO_2_ structure was further prepared by coating 120 nm Au NPs with a thin layer of silica (∼2 nm), which can prevent the interaction between 120 nm Au NPs and analytical targets (Fig. 1c).^40,41^ Then, Au_100_ NPs, Au_90_Cu_10_ NPs, and Au_67_Cu_33_ NPs were deposited on Au@SiO_2_, forming Au@SiO_2_@Au_100_, Au@SiO_2_@Au_90_Cu_10_, and Au@SiO_2_@Au_67_Cu_33_ catalysts, respectively. The UV-vis extinction spectrum results showed that the peak of plasmon resonance was shifted from 604 cm^−1^ to 625 cm^−1^ after depositing nanocatalysts on Au@SiO_2_, indicating the successful decoration of Au@SiO_2_ with nanocatalysts (Fig. S5†). Fig. 1d shows the TEM characterization and elemental mapping images of a single Au@SiO_2_@Au_67_Cu_33_ catalyst with a 120 nm Au core and many Au_67_Cu_33_ NPs as the satellites. Therefore, the Raman signal from surface species on Au_100_ NPs, Au_90_Cu_10_ NPs, or Au_67_Cu_33_ NPs can be amplified by using a SERS-active Au core and detected using *in situ* spectra. These results make sure that the hydrogenation process of *p*-NTP on Au@SiO_2_@Au_100_, Au@SiO_2_@Au_90_Cu_10_, or Au@SiO_2_@Au_67_Cu_33_ catalysts can be investigated by using *in situ* SERS (Fig. 1e).

**Fig. 2:**
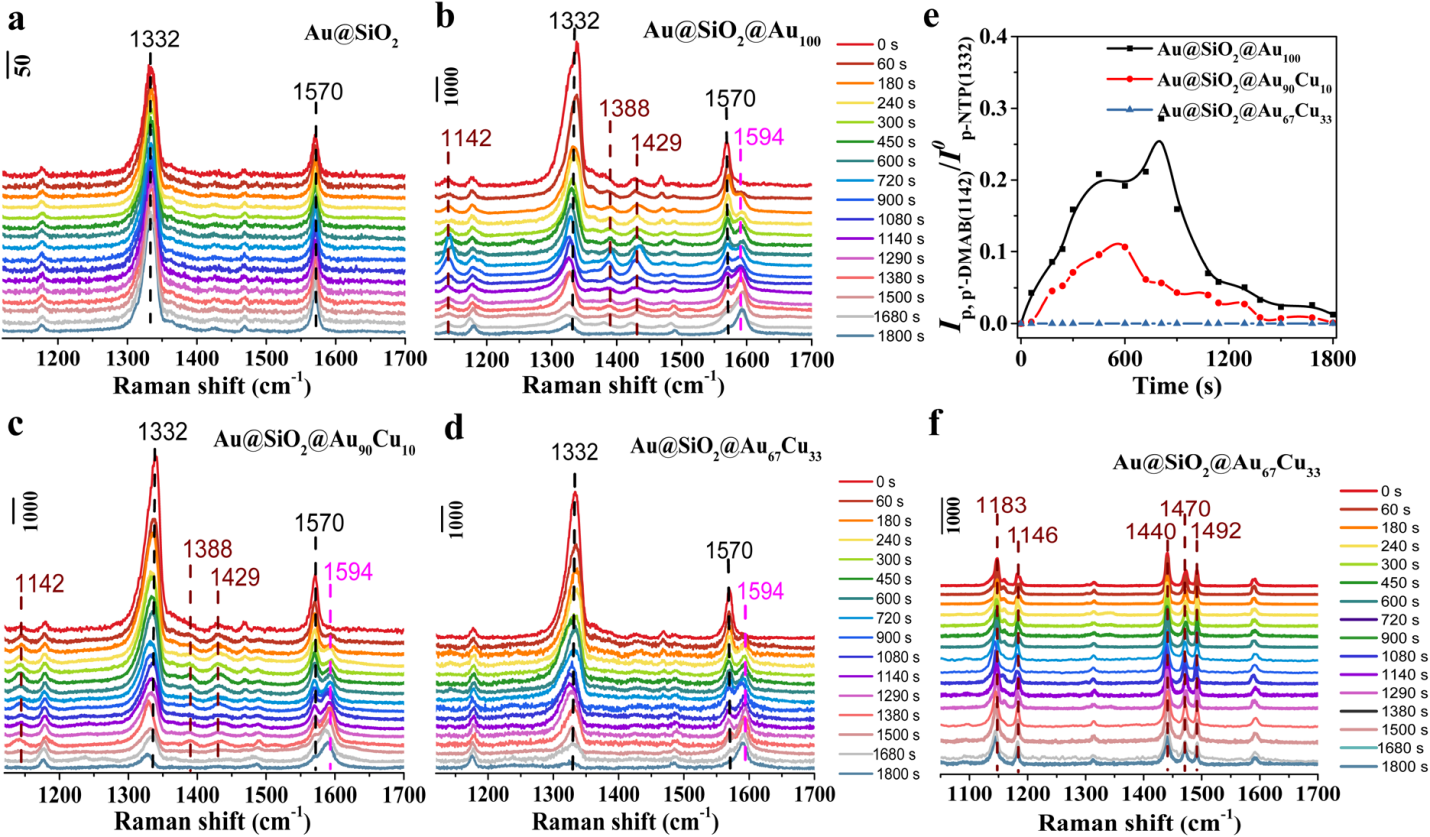
*In situ* SERS spectra of the hydrogenation reaction of *p*-NTP in 8.5 × 10^−3^ M NaBH_4_ aqueous solution on (a) Au@SiO_2_, (b) Au@SiO_2_@Au_100_, (c) Au@SiO_2_@Au_90_Cu_10_, and (d) Au@SiO_2_@Au_67_Cu_33_. (e) Time-dependent intensity variation curves of the Raman peaks for *p*,*p*′-DMAB (at ∼1142 cm^−1^) on Au@SiO_2_@Au_100_, Au@SiO_2_@Au_90_Cu_10_, and Au@SiO_2_@Au_67_Cu_33_. (f) *In situ* SERS spectra of the hydrogenation reaction of DMAB in 8.5 × 10^−3^ M NaBH_4_ aqueous solution on Au@SiO_2_@Au_67_Cu_33_. Note that the SNR of the SERS signal between different catalysts is similar (Table S1†), making sure that the comparison of SERS spectra between different catalysts is valid.

First, to track the hydrogenation process of *p*-NTP on single-component Au_100_ NPs, *in situ* SERS was used to track the hydrogenation process on a Au@SiO_2_@Au_100_ catalyst with sodium borohydride (NaBH_4_) as a reduction agent. Before the reaction, the catalyst was immersed in the *p*-NTP aqueous solution overnight, ensuring the adsorption of the *p*-NTP molecule at the catalyst surfaces with saturation. Then, the reaction started once the catalyst adsorbing *p*-NTP came into contact with the NaBH_4_ aqueous solution. During the reaction process, *p*-NTP can be reduced by the active hydrogen species (H*), which are formed from the dissociation activation of NaBH_4_ on the catalyst surface.^42^ And *in situ* SERS spectra of the hydrogenation reaction of *p*-NTP on different catalysts were collected at a certain time interval. First, the hydrogenation process of *p*-NTP on Au@SiO_2_ was first studied by *in situ* SERS and is shown in Fig. 2a. At 0 s, the Raman peaks at 1332 cm^−1^ and 1570 cm^−1^, assigned to the NO_2_-stretching and C–C-stretching band in the benzene ring of substrate *p*-NTP,^41^ were observed obviously, indicating the successful adsorption of *p*-NTP on Au@SiO_2_. However, these peaks did not show any change during the reaction process even on extending the reaction time to 1800 s. And we did not detect any new Raman peaks, indicating that Au@SiO_2_ is inert in catalyzing the hydrogenation of *p*-NTP. Thus, Au@SiO_2_ acted merely as a platform to enhance the Raman signal of surface species. However, for the Au@SiO_2_@Au_100_ catalyst, the intensity of Raman peaks associated with substrate *p*-NTP at 1332 cm^−1^ and 1570 cm^−1^, was decreased gradually as the reaction proceeded from 0 s to 1800 s as seen in the *in situ* SERS spectra (Fig. 2b). Meanwhile, a new peak centered at 1594 cm^−1^, which was assigned to the stretching coordinate of the benzene ring of product *p*-ATP,^41,47^ was detected and the intensity of this new peak was increased gradually with the reaction time. These results indicate that *p*-NTP can be hydrogenated to *p*-ATP on Au@Au_100_. Besides, in addition to the observation of the peaks associated with substrate *p*-NTP and product *p*-ATP, three new peaks located at 1142 cm^−1^, 1388 cm^−1^ and 1429 cm^−1^ were detected in the *in situ* SERS spectra recorded on Au@SiO_2_@Au_100_. These three peaks were assigned to the C–N stretching and N=N stretching vibrational band of coupling intermediate product *p*,*p*′-dimercaptoazobenzene (*p*,*p*′-DMAB).^41^ Interestingly, the intensity of peaks assigned to *p*,*p*′-DMAB was first increased and then decreased until it disappeared at 1800 s, meaning *p*,*p*′-DMAB was the intermediate species during the hydrogenation of *p*-NTP to *p*-ATP on the Au@SiO_2_@Au_100_ catalyst. The above results confirmed that *p*,*p*′-DMAB was directly involved in the *p*-NTP hydrogenation process on Au_100_ NPs, indicating the presence of the coupling route synthesizing *p*-ATP from *p*-NTP. The control experiment showed that there was no reaction without NaBH_4_, indicating that NaBH_4_ acted as the reduction agent in the hydrogenation of *p*-NTP (Fig. S6†).

Then, the hydrogenation of *p*-NTP on Au@SiO_2_@Au_90_Cu_10_ (Fig. 2c) and Au@SiO_2_@Au_67_Cu_33_ (Fig. 2d) catalysts was further studied by *in situ* SERS with the same measurement conditions as the Au@SiO_2_@Au_100_ catalyst. *In situ* SERS spectra obtained on Au@SiO_2_@Au_90_Cu_10_ were similar to that on Au@SiO_2_@Au_100_ except for the observation of the lower intensity of peaks assigned to *p*,*p*′-DMAB during the detection time of 1800 s (Fig. 2c). On further increasing the mole percentages of Cu from 10 of Au@SiO_2_@Au_90_Cu_10_ to 33 of Au@SiO_2_@Au_67_Cu_33_, the peaks assigned to *p*,*p*′-DMAB were not detected in the *in situ* SERS spectra recorded on Au@SiO_2_@Au_67_Cu_33_. To compare the discrepancy of chemical behaviors on Au@SiO_2_@Au_100_, Au@SiO_2_@Au_90_Cu_10_, and Au@SiO_2_@Au_67_Cu_33_ during the hydrogenation reaction of *p*-NTP, the initial coverage of *p*-NTP (Fig. S7†) and time-dependent intensity variation curve of the Raman peaks for *p*,*p*′-DMAB (at ∼1142 cm^−1^) on the three catalysts were plotted (Fig. 2e). Based on the time-dependent coverage variation curves of *p*-NTP on the three catalysts, we found that the coverage of *p*-NTP on the three different catalysts decreased when the reaction started. And the coverage of *p*-NTP on Au@SiO_2_@Au_67_Cu_33_ showed a faster decrease rate, indicating that Cu doping can improve the hydrogenation of *p*-NTP. The time-dependent intensity variation curve of the Raman peaks for *p*,*p*′-DMAB results showed that the peak intensity of *p*,*p*′-DMAB decreased in the order of Au@SiO_2_@Au_100_ > Au@SiO_2_@Au_90_Cu_10_ > Au@SiO_2_@Au_67_Cu_33_, proposing that the Cu doping can inhibit the formation of coupling product *p*,*p*′-DMAB. And *p*,*p*′-DMAB might not the reaction intermediate during the hydrogenation of *p*-NTP to *p*-ATP over Au@SiO_2_@Au_67_Cu_33_. A control experiment, tracking the hydrogenation of azobenzene (DMAB) on Au@SiO_2_@Au_67_Cu_33_ by *in situ* SERS, further confirmed this deduction. In the *in situ* SERS spectra, five peaks located at 1183, 1146, 1440, 1470, and 1492 cm^−1^, were observed obviously, which are assigned to the C–N and N=N stretching modes of DMAB (Fig. 2f). And there was no change for the peaks assigned to DMAB with reaction time,^48^ confirming that *p*,*p*′-DMAB was not the reaction intermediate during the hydrogenation of *p*-NTP to *p*-ATP over Au@SiO_2_@Au_67_Cu_33_. These results presented the direct spectral evidence of route regulation from the coupling route on Au_100_ NPs to the direct route on Au_67_Cu_33_ NPs for hydrogenation of *p*-NTP to *p*-ATP at a molecular level. That said, the reaction route can be regulated to the direct route owing to a higher Cu concentration in Au_67_Cu_33_ NPs.

**Fig. 3:**
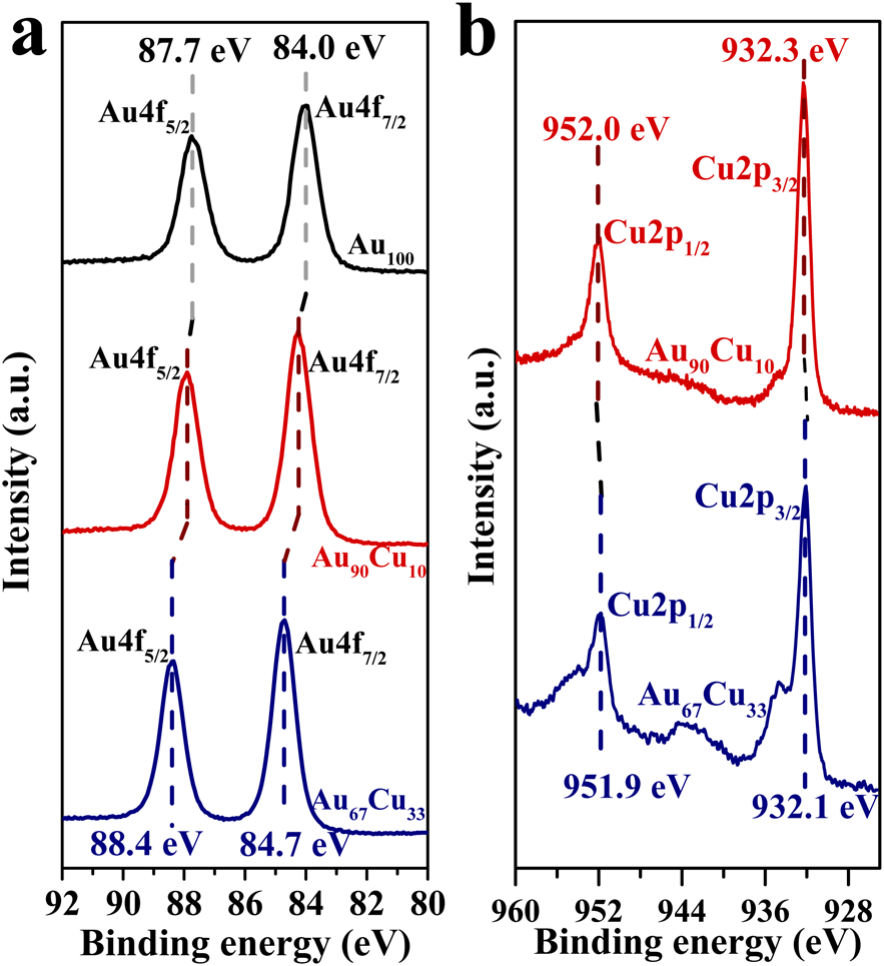
(a) Au 4f XPS spectra of Au_100_, Au_90_Cu_10_ and Au_67_Cu_33_ NPs. (b) Cu 2p XPS spectra of Au_90_Cu_10_ and Au_67_Cu_33_ NPs.

The regulation of the reaction route from the coupling route to the direct route by alloying Cu in Au may have a great relationship with the change in its electronic structure.^49,50^ Thus, the XPS characterization studies for Au_100_ NPs, Au_90_Cu_10_ NPs, and Au_67_Cu_33_ NPs were conducted and are shown in Fig. 3. Two peaks at binding energies of 84.0 and 87.7 eV were observed for Au_100_ NPs (Fig. 3a), in accordance with the values for zerovalent Au 4f_7/2_ and Au 4f_5/2_ as previously reported.^51^ However, a slight positive shift of the binding energies of Au 4f_7/2_ and Au 4f_5/2_ was observed from the XPS characterization result of Au_90_Cu_10_ NPs. With an increase in the Cu concentration, the binding energies of both Au 4f_7/2_ and Au 4f_5/2_ for Au_67_Cu_33_ NPs were further positively shifted to 84.7 and 88.4 eV, indicating the decrease in the electron density of Au,^52^ whereas the electron density of Cu increased with the increase in Cu concentration based on the Cu 2p XPS spectra of Au_90_Cu_10_ and Au_67_Cu_33_ NPs (Fig. 3b). This clearly indicated that electrons were transferred from Au to Cu when these elements form alloy NPs. And a higher Cu concentration in Au_100−*x*_Cu_*x*_ NPs leads to an increase in the transfer number of electrons from Au to Cu.

**Fig. 4:**
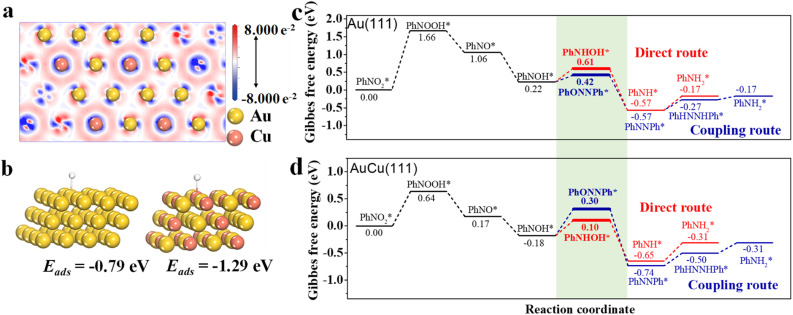
(a) Top view of the difference in charge density for AuCu(111), in which blue and red regions indicate electron accumulation and depletion, respectively. (b) Adsorption model of H species on Au(111) and AuCu(111) and their corresponding adsorption energies. Calculated relative energy change for the reaction route on (c) Au(111) and (d) AuCu(111).

To further understand the critical role of Cu in route regulation from the coupling route on Au_100_ NPs to the direct route on Au_67_Cu_33_ NPs for hydrogenation of *p*-NTP to *p*-ATP, density functional theory (DFT) calculations were performed based on Au(111) and AuCu(111). To reduce the complexity of computation, we focused on the hydrogenation process of nitrobenzene (PhNO_2_) to phenylamine (PhNH_2_) during DFT calculations. First, the charge distribution of AuCu(111) was analyzed through the charge density difference (Fig. 4a). We found that the charge accumulation is shown as the blue region close to Cu atoms, and the charge depletion is shown as the red region close to Au atoms, meaning that electrons can transfer from Au to Cu. This result is in accordance with the results from XPS characterization studies. The absorption energy of H species, an important active species during hydrogenation of PhNO_2_, was also calculated on the surface of Au(111) and AuCu(111) here (Fig. 4b). The absorption energy of H species on Au(111) was calculated to be −0.79 eV. However, for AuCu(111), H species is favored to adsorb on the Cu atom with a stronger absorption energy of −1.29 eV. This result proved that it is easier for the active Cu–H species with stronger absorption energy to be formed on AuCu(111). Additionally, the adsorption energies of PhNO_2_ on AuCu(111) and Au(111) were further calculated, respectively. The results showed that the adsorption energy of PhNO_2_ on the surface of AuCu(111) (−1.37 eV) was stronger than that of Au(111) (−1.05 eV) (Fig. S8†). The stronger adsorption energy of PhNO_2_ on AuCu(111) can suppress the formation of coupling products, favoring the formation of PhNH_2_ with a direct route.^8^

To deeply gain insight into the origin of the reaction route regulation by doping Cu in Au for the hydrogenation of nitroaromatics, DFT calculations were further carried out on the reaction steps on Au(111) (Fig. 4c) and AuCu(111) (Fig. 4d), respectively. After being adsorbed on the Au(111) or AuCu(111) surface, *PhNO_2_ formed PhNOOH* by combining with H*, which then quickly dissociated to form PhNO* and OH*. PhNO* further combined with H* to generate PhNOH*. There are two reaction routes for the further transformation of PhNOH*. (i) PhNOH* can couple with PhNO*, forming PhON = NPh* and facilitating the formation of PhNH_2_* *via* the coupling route; (ii) PhNOH* can undergo hydrogenation to form phenylhydroxylamine (PhNHOH*), which favors the occurrence of a direct route to form PhNH_2_*. However, the Gibbs free energies of the same intermediates on Au(111) and the AuCu(111) surface are different, leading to the existence of different reaction routes between Au(111) and the AuCu(111) surface. For the Au(111) surface, the energy for PhNOH* hydrogenation to PhNHOH* is 0.61 eV, which is higher than that for the coupling reaction between PhNOH* and PhNO* (0.42 eV) (Fig. 4c). This result shows that PhNOH* favors to couple with PhNO*, leading to the appearance of a coupling route at Au(111). For AuCu(111), the formation energy of PhNHOH* (0.10 eV) is much lower than that of coupling intermediate PhON = NPh* (0.30 eV), indicating that AuCu(111) favors the direct route to form PhNH_2_* by facilitating the formation of PhNHOH* (Fig. 4d). This unambiguously confirms that AuCu(111) can effectively reduce the energy barrier of PhNOH* hydrogenation to PhNHOH*, resulting in the favoring of the occurrence of a direct route on the surface of AuCu(111). In addition, the binding geometries of the reaction intermediates on Au(111) or the AuCu(111) surface, are shown in Fig. S9.†

Based on the results obtained from *in situ* SERS experiments, XPS characterization studies, and theoretical calculations, the route regulation mechanism mediated by bimetallic Au_67_Cu_33_ NPs for the catalytic PhNO_2_ hydrogenation reaction was proposed. First, NaBH_4_ is activated on the catalyst surface, forming active hydrogen species. The Cu–H species formed on Au_67_Cu_33_ NPs has stronger adsorption energy than Au–H species on Au_100_ NPs. For Au_100_ NPs, PhNOH* favors to couple with PhNO*, forming PhON = NPh* and promoting the occurrence of the coupling route. However, the formed active Cu–H species on Au_67_Cu_33_ NPs with stronger adsorption energy can promote the direct hydrogenation of PhNOH* to form PhNHOH*, favoring the appearance of a direct route.

## Conclusions

In summary, we used *in situ* SERS to systematically explore the hydrogenation process of *p*-NTP to *p*-ATP on Au@SiO_2_@Au_100_ as well as bimetallic Au@SiO_2_@Au_90_Cu_10_ and Au@SiO_2_@Au_67_Cu_33_ and obtained the direct spectral evidence of the route regulation mediated by Au_67_Cu_33_ NPs. We found that during the hydrogenation process of *p*-NTP to *p*-ATP, *p*,*p*′-DMAB species was detected and the detected *p*,*p*′-DMAB species increased first and then decreased on the Au_100_ NPs, indicating the presence of the coupling route to from *p*-ATP. However, *p*-ATP was the sole product on Au@SiO_2_@Au_67_Cu_33_ NPs, meaning the existence of a direct route during the hydrogenation process of *p*-NTP to *p*-ATP. By combining XPS and DFT calculations, we found that Cu doping can favor the formation of active Cu–H species owing to the electron accumulation of Cu, which can promote the formation of PhNHOH* and lead to regulation in the pathway to the direct route on Au_67_Cu_33_ NPs. Our study provides direct spectral evidence demonstrating the critical role of Cu in the route regulation for hydrogenation reactions of nitroaromatics on bimetallic Au_67_Cu_33_ NPs at a molecular level and clarifies the route regulation mechanism. This result has significant implications for multimetallic alloy nanocatalyst mediated reaction mechanisms and helps to guide the rational design of multimetallic alloy catalysts for catalytic hydrogenation reactions.

## Data availability

The experimental or computational data associated with this article are placed in the ESI.†

## Author contributions

The manuscript was written through contributions of all authors. Jinghua An, Lu Li and Bo Tang designed the research, Xiaoxiao Li, Ze Gao and Yaoying Cheng performed the research, Xiaoxiao Li, Ze Gao, Jinghua An, Chang Xu, Simin Li and Lu Li analyzed the data; Xiaoxiao Li, Jinghua An, Lu Li and Bo Tang wrote the paper. All authors have given approval to the final version of the manuscript.

## Conflicts of interest

There are no conflicts to declare.

## Supplementary Material

SC-014-D2SC06808G-s001
