# Supplementary material for: In situ SERS reveals the route regulation mechanism mediated by bimetallic alloy nanocatalysts for the catalytic hydrogenation reaction
Source: Chem Sci. 2023 Feb 27;14(13):3554–61. doi: 10.1039/d2sc06808g (PMC10055342; doi:10.1039/d2sc06808g)
Supplement: SC-014-D2SC06808G-s001 [file SC-014-D2SC06808G-s001.pdf]

Electronic Supplementary Material (ESI) for Chemical Science.
This journal is © The Royal Society of Chemistry 2023

In situ SERS reveals route regulation mechanism mediated by bimetallic alloy nanocatalysts for catalytic hydrogenation reaction

Xiaoxiao Li,[#] *Jinghua An,*[#] *Ze Gao, Chang Xu, Yaoying Cheng, Simin Li, Lu Li,* and Bo Tang**

College of Chemistry, Chemical Engineering and Materials Science, Key Laboratory of Molecular and Nano Probes, Ministry of Education, Collaborative Innovation Center of Functionalized Probes for Chemical Imaging in University of Shandong, Institute of Molecular and Nano Science, Shandong Normal University, Jinan, 250014, China.

1. Materials

$HAuCl_4 \cdot 3H_2O$, 1,2-Hexadecanediol, oleylamine, copper(II) acetylacetonate, sodium silicate solution were purchased from Sigma Aldrich. Trisodium citrate, (3-aminopropyl) trimethoxysilane (APTMS), 1-octadecene (90%), oleic acid were purchased from Alfa-Aesar. Azobenzene (DMAB), Sodium borohydride ($NaBH_4$), 3-mercaptopropyltrimethoxysilane (MPTMS), and para-nitrothiophenol (p-NTP) were purchased from Shanghai Aladdin Bio-Chem Technology (Shanghai, China). Hexanes, ethyl alcohol, ammonium hydroxide, and acetone were purchased from Sinopharm Chemical Reagent (Shanghai, China). Deionized water was provided by Hangzhou Wahaha Group (Zhejiang, China). All reagents were analytically pure.

2. Material characterizations.

The transmission electron microscopy (TEM) was carried out on a Hitachi HT7700 transmission electron microscope (JEOL Ltd, Japan) and the samples were prepared *via* carbon-coated copper grids. The UV-vis extinction spectra were obtained with a Hitachi U-3010 UV-vis spectrophotometer (JEOL Ltd, Japan). Detailly, 10 mM $PhNO_2$ ethanol solution (10 μL) was added to 1 mL $Au@SiO_2@Au100$, $Au@SiO_2@Au_{90}Cu_{10}$ and $Au@SiO_2@Au_{67}Cu_{33}$ colloidal suspensions for UV-vis extinction spectra detection. The X-ray powder diffraction (XRD) patterns of the catalysts were performed on a Smart Lab Se (Rigaku, Japan). The X-ray photoelectron spectroscopy (XPS) of the catalysts was obtained with an ESCALAB 250Xi (Thermo Fisher Scientific, America), using an incident Al Kα radiation of 1486 eV. The high-resolution transmission electron microscopy (HRTEM) was performed on a JEOL JEM-2100 electron microscope operating with a LaB6 cathode and an acceleration voltage of 200 kV. Compositional maps were obtained with energy-dispersive X-ray spectroscopy (EDX) using four large-solid-angle symmetrical Si drift detectors.

3. Synthesis of 120 nm Au NPs.

The Au NPs with a diameter of 120 nm were prepared *via* the seed growth method.[1] Typically, 100 mL 2.2 mM sodium citrate aqueous solution was heated to boiling under vigorous stirring. After that, 1 mL 25 mM chloroauric acid aqueous solution

($HAuCl_4$) was rapidly added to the boiling solution. The color of the mixture solution changed from pale yellow to bluish-gray and then to burgundy. Then the solution temperature was slowly cooled to 90 °C under continuous stirring. At the moment, 1 mL 25 mM $HAuCl_4$ solution was added to the above system quickly and kept for 30 min. After 30 min, 1 mL 25 mM $HAuCl_4$ solution was added to the above system quickly and kept for another 30 min. Then, 55 mL of the above solution was named as g_x (x=0−5) and was used as a seed solution to obtain Au NPs of a larger size. 53 mL water and 2 mL 60 mM sodium citrate aqueous were added to the 55 mL seed solution. Then, 1 mL $HAuCl_4$ solution (25 mM) was injected into the above solution quickly, 30 min later, 1 mL $HAuCl_4$ solution (25 mM) was injected into the above solution quickly and kept for another 30 min. The above steps were repeated five times until the diameter of the final product (g_x) was about 120 nm.

4. Synthesis of Au@SiO_2 substrate.

The Au@SiO_2 was prepared based on the literature report.[2] Typically, 0.4 mL 1 mM (3-aminopropyl) trimethoxysilane (APTMS) was added to the as-prepared 120 nm Au NPs at room temperature under vigorous stirring for 3 min. Then, 3.2 mL 0.54% $NaSiO_3$ aqueous solution was introduced to the above solution under intense stirring at room temperature for 3 min. The mixture solution was heated to 98 °C for 20 min to accelerate the coating of the ~3 nm SiO_2 shell on the surface of 120 nm Au NPs. After that, the mixture was cooled rapidly in an ice bath to prevent the further growth of SiO_2 shell. Eventually, the Au@SiO_2 substrate was obtained by centrifugation at 4000 rpm for 5 min. Finally, the obtained Au@SiO_2 were redispersed in 1 mL ethanol for further experiments.

5. Synthesis of 13 nm Au_{100} NPs.

The 13 nm Au_{100} NPs were prepared by the sodium citrate reduction method.[3] Firstly, 100 mL 1 mM $HAuCl_4$ aqueous solution was heated to boiling under constantly stirring. After that, 10 mL 38.8 mM sodium citrate solution was added to the boiling solution quickly. The color of the above-mixed solution changed from pale yellow to burgundy within 3 minutes, indicating the formation of Au NPs. The solution was maintained for 30 min at a boiling state under vigorous stirring to ensure complete reduction and slowly cooled to room temperature with continuous stirring. The obtained solution containing Au NPs was stored in the fridge at 4 °C for further

experiments. TEM characterization confirmed that the average size of catalytic active Au NPs was~13 nm. And the 13 nm Au NPs were named Au_{100} NPs.

6. Synthesis of 13 nm $Au_{90}Cu_{10}$ NPs and 13 nm $Au_{67}Cu_{33}$ NPs.

The 13 nm $Au_{90}Cu_{10}$ NPs and $Au_{67}Cu_{33}$ NPs were prepared by the sodium citrate reduction method.[4] In the synthesis of 13 nm $Au_{67}Cu_{33}$ NPs, 600 μL oleylamine, 800 μL oleic acid, and 5 mL 1-octadecene were added into a 100 mL round bottom flask containing 45 mg $HAuCl_4 \cdot 3H_2O$, 109 mg copper acetylacetonate, and 100 mg 1,2-hexadecanediol. Then, the mixture solution was heated to 120 °C under vigorous stirring for 35 min and then heated to 245 °C, maintaining for 1 h. The solution was then cooled to room temperature within 40 min. *Note: All above reactions were carried out on a Schlenk line using standard air-free techniques.* The crude solid containing $Au_{67}Cu_{33}$ NPs were precipitated by adding a 20 mL mixture solution of acetone and hexane (3:1) and separated by centrifugation at 12000 rpm for 10 min. After that, the solid was redispersed in a 40 mL hexane solution containing 1wt % oleylamine and 1wt % oleic acid. The dispersion was then centrifugated at 3000 rpm for 5 min. The under solution containing larger particles or aggregates was abandoned, leaving 20 mL upper solution containing 13 nm $Au_{67}Cu_{33}$ NPs. Finally, 13 nm $Au_{67}Cu_{33}$ NPs were separated by centrifugation at 12000 rpm for 10 min and redispersed in 7 mL hexane for further experiments. The procedure synthesizing 13 nm $Au_{90}Cu_{10}$ is similar with that of 13 nm $Au_{67}Cu_{33}$. The difference is that the input of acetyl copper is 28 mg.

7. Synthesis of $Au@SiO_2@Au_{100}$, $Au@SiO_2@Au_{90}Cu_{10}$, and $Au@SiO_2@Au_{67}Cu_{33}$.

Firstly, 1 mL $Au@SiO_2$ ethanol suspension was mixed with 5 μL of MPTMS ethanol solution (10 wt%) and 50 μL ammonium hydroxide aqueous solution (25 wt %) at room temperature. Then, the mixture solution was shaken for 40 min, insuring the modification of positive charge on the surface of $Au@SiO_2$. The $Au@SiO_2$ with positive charge was then separated by centrifugation at 4000 rpm for 5 min and resuspended in 1 mL ethanol. After that, the $Au@SiO_2$ with a positive charge was added into 1 mL as-prepared dispersion of 13 nm Au_{100} NPs, 13 nm $Au_{90}Cu_{10}$ NPs or 13 nm $Au_{67}Cu_{33}$ NPs to react for 12 h under stirring, ensuring the assembly of nanocatalysts on $Au@SiO_2$. Then, the $Au@SiO_2@Au_{100}$, $Au@SiO_2@Au_{90}Cu_{10}$, and

$Au@SiO_2@Au_{67}Cu_{33}$ were obtained by washing with ethanol to remove free 13 nm Au_{100} NPs, 13 nm $Au_{90}Cu_{10}$ NPs or 13 nm $Au_{67}Cu_{33}$ NPs. Finally, the $Au@SiO_2@Au_{100}$, $Au@SiO_2@Au_{90}Cu_{10}$, or $Au@SiO_2@Au_{67}Cu_{33}$ catalysts were redispersed in 1 mL ethanol for further experiments.

8. *In situ* SERS measurements.

Firstly, 10 μL 10 mM p-NTP ethanol solution was added to 1 mL $Au@SiO_2@Au_{67}Cu_{33}$ ethanol solution. The mixture was shaken overnight at room temperature, ensuring the adsorption of p-NTP molecules on the surface of $Au@SiO_2@Au_{67}Cu_{33}$ catalyst.[5] Then, the suspension was washed twice with ethanol to remove the free p-NTP, obtaining a molecule layer of p-NTP on the surface of $Au@SiO_2@Au_{67}Cu_{33}$.[6, 7] The $Au@SiO_2@Au_{67}Cu_{33}$ adsorbed p-NTP was redispersed in 200 μL ethanol solution. *In situ* SERS spectrum was collected once the addition of $NaBH_4$ aqueous solution into the above mixture. Each SERS spectrum was collected at a certain time interval at 25 °C using He-Ne laser operating at λ = 633 nm and a 50× long objective lens. The laser output power was controlled at 10 mW. The procedure of *in situ* SERS measurement over $Au@SiO_2$, $Au@SiO_2@Au_{100}$, $Au@SiO_2@Au_{90}Cu_{10}$ is similar to that of $Au@SiO_2@Au_{67}Cu_{33}$ catalysts. The exposure time for each SERS spectrum is 5 s. The laser beam size on the sample is 1 μm and the corresponding laser intensity is 1.2 mW/μm^2.

9. Computational method.

We have employed the Vienna Ab Initio Package (VASP)[8, 9] to perform all spin-polarization DFT calculations within the generalized gradient approximation (GGA) using the Perdew-Burke-Ernzerhof (PBE) formulation.[10] In addition, the adsorption model was optimized by Newton's method of second order convergence (IBRION=1 and POTIM=0.2). We have chosen the projected augmented wave (PAW) potentials[11, 12] to describe the ionic cores and take valence electrons into account using a plane-wave basis set with a kinetic energy cutoff of 520 eV. Partial occupancies of the Kohn−Sham orbits were allowed to use the Gaussian smearing method and a width of 0.05 eV. The electronic energy was considered self-consistent when the energy change was smaller than 10^{-5} eV. Geometry optimization was considered convergent when the energy change was smaller than 0.03 eV·Å^{-1}. The equilibrium lattice constants of AuCu(111) and Au(111) surface were optimized with the lattice

parameters (a=11.7982Å, b=11.7982Å, c=27.2249Å). During structural optimizations, a 3×3×1 k-point grid in the Brillouin zone was used for k-point sampling, and the bottom two atomic layers were fixed while the top two were allowed to relax. Finally, the adsorption energies (E_{ads}) were calculated as:

$$E_{ads}= E_{ad/sub} - E_{ad} - E_{sub}$$

Where $E_{ad/sub}$, E_{ad}, and E_{sub} are the total energies of the optimized adsorbate/substrate system, the adsorbate in the gas phase, and the clean substrate, respectively. The Brillouin zone integral uses the surface structures of 3×3×1 monk horst pack K point sampling for structures. The free energy (ΔG) for the elemental reaction step was calculated as:

$$\Delta G = \Delta E + \Delta EZPE - T\Delta S$$

where ΔE is the difference between the total energy, ΔEZPE and ΔS are the differences in the zero-point energy and the change of entropy, T is the temperature (T = 300 K in this work).

10. Calculated procedure for SNR of SERS signal for different catalysts.

The SNR of SERS signal for different catalysts were calculated according to the literature report.[13] Signal-to-Noise-Ratio (SNR) is defined by:

$$\mathrm{SNR}=10\lg\frac{P_{signal}}{P_{noise}}$$

Where P_{signal} and P_{noise} are the power of signal and the power of noise.

In order to calculate the SNRs of SERS signal for different catalysts, five different Raman spectra were obtained on the same catalyst without the addition of $NaBH_4$. Following is the detailed procedure:

i. The signal power (P_{signal}) was calculated according to the equation: $P_{signal} = \frac{1}{5}\sum_{i=1}^{5} I_{signal}^{2}$. The value of I_{signal} of the five different Raman spectra is obtained by subtracting the value of baseline from original Raman signal intensity at 1570 cm^{-1}.

ii. The noise power (P_{noise}) was calculated according to the equation: $P_{noise} = \frac{1}{5}\sum_{i=1}^{5} I_{noise}^{2}$. In order to obtain I_{noise} of the five different Raman spectra, the average signal intensity at 1570 cm^{-1} should be first calculated and the corresponding value of I_{noise} is obtained by subtracting average peak intensity from the original Raman signal intensity at 1570 cm^{-1}.

iii. The SNR of each catalyst is calculated by $SNR=10lg(\frac{P_{signal}}{P_{noise}})$

The result is given in Table S1.

Table S1. **The SNR of different catalysts.**

Catalysts	$Au@SiO_2@Au_{100}$	$Au@SiO_2@Au_{90}Cu_{10}$	$Au@SiO_2@Au_{67}Cu_{33}$
SNR	21.08	25.56	21.96

Table S2. ICP-MS results

Nanocatalysts	Au (mg/L)	Cu (mg/L)	Mole ratios of Au to Cu
$Au_{90}Cu_{10}$	0.222	0.008	90:10
$Au_{67}Cu_{33}$	0.280	0.047	67:33

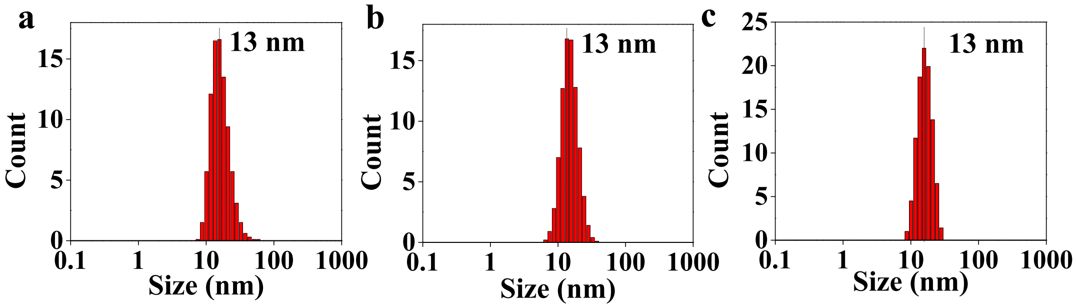

Figure S1. The particle size distribution of (a) Au_{100} NPs, (b) bimetallic $Au_{90}Cu_{10}$ NPs and (c) $Au_{67}Cu_{33}$ NPs.

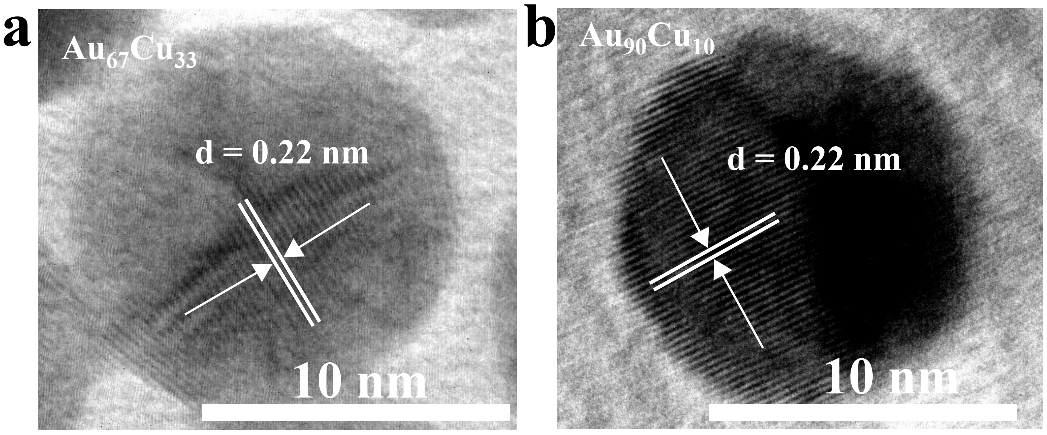

Figure S2. HRTEM image of $Au_{67}Cu_{33}$ NPs and $Au_{90}Cu_{10}$ NPs.

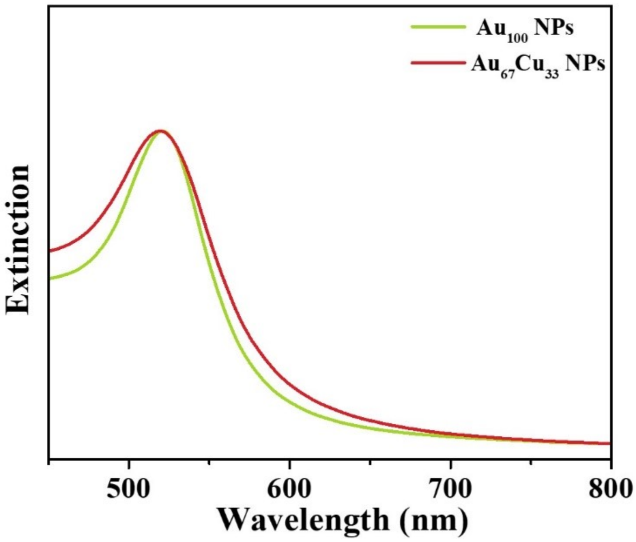

Figure S3. UV-vis extinction spectra of Au_{100} and $Au_{67}Cu_{33}$ NPs.

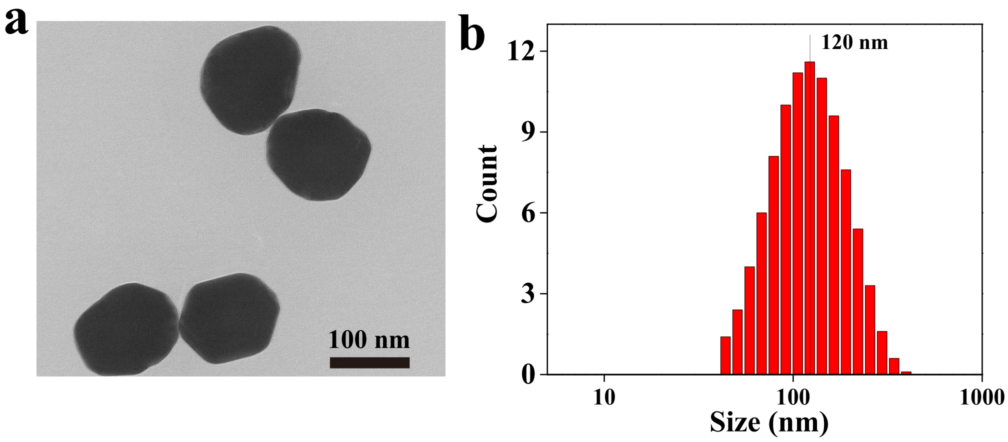

Figure S4. (a) TEM image of SERS-active Au nanoparticles with a diameter of 120 nm. (b) The size distribution of SERS-active 120 nm Au nanoparticles.

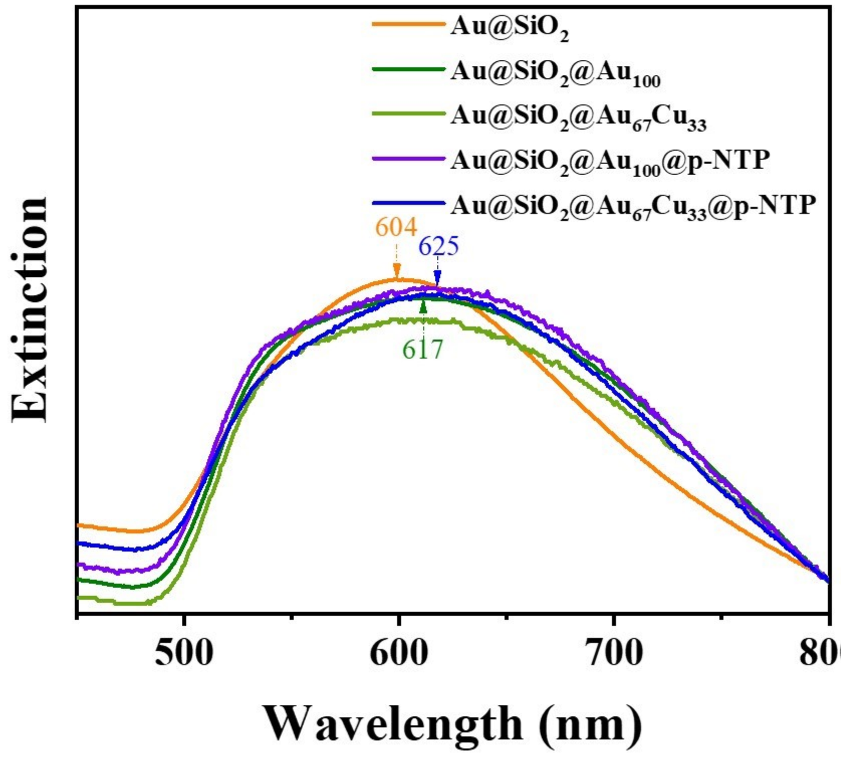

Figure S5. UV-vis extinction spectra of $Au@SiO_2$, $Au@SiO_2@Au_{100}$, $Au@SiO_2@Au_{67}Cu_{33}$, $Au@SiO_2@Au_{100}$@p-NTP and $Au@SiO_2@Au_{67}Cu_{33}$@p-NTP.

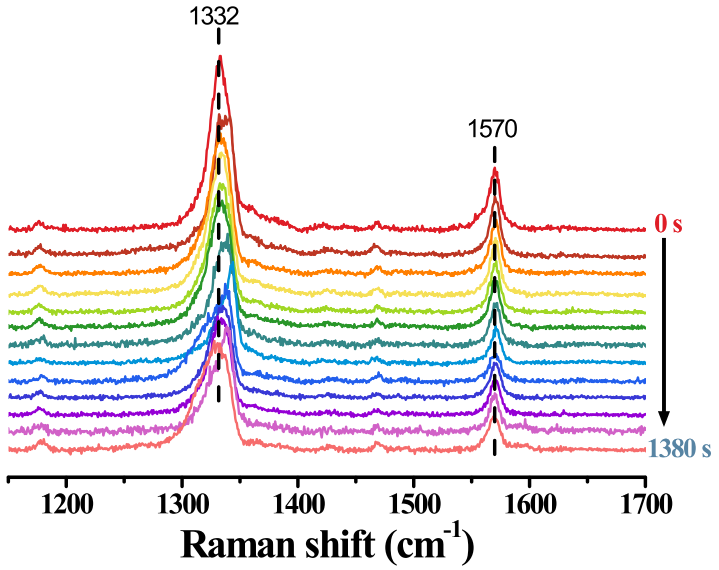

Figure S6. *In situ* SERS spectra of p-NTP on $Au@SiO_2@Au_{100}$ without $NaBH_4$.

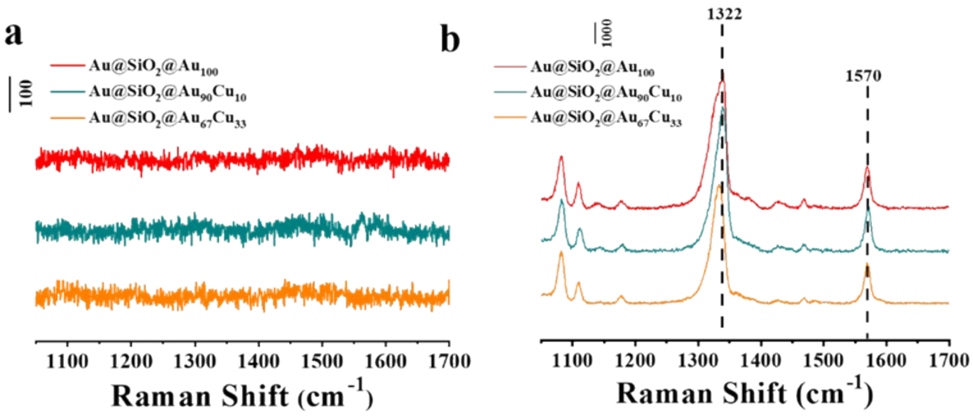

Figure S7. The SERS intensities of (a) background and (b) the initial Raman spectra of p-NTP molecule on the three types $Au@SiO_2@Au_xCu_x$ substrates.

The initial surface coverage of p-NTP was proportional to the initial Raman intensity of the peak at 1570 cm^{-1} for p-NTP molecules on the three different catalysts. The result shows that the initial Raman intensity of the peak at 1570 cm^{-1} for p-NTP molecules is similar, indicating that the initial surface coverage of p-NTP is similar.

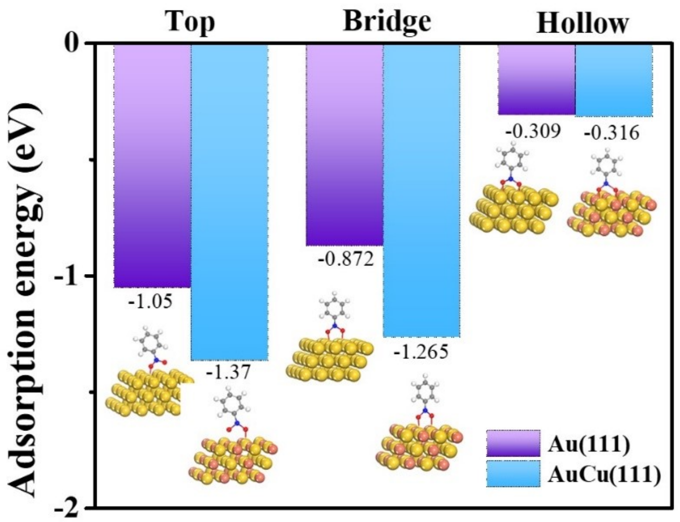

Figure S8. The binding geometries and the corresponding adsorption energies of PhNO2 on Au(111) and AuCu(111) surface.

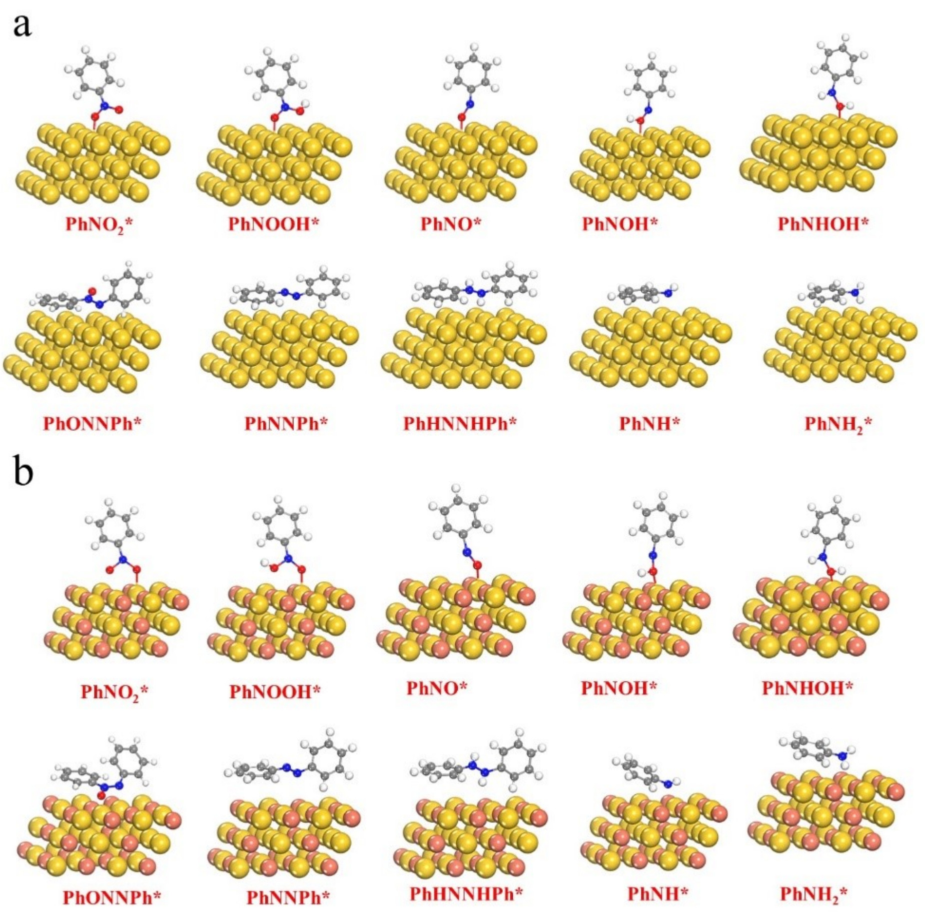

Figure S9. The binding geometries of the reaction intermediates on (a) Au(111) and (b) AuCu(111) surface.

Notes and references

1. Bastus, N. G.; Comenge, J.; Puntes, V., Kinetically controlled seeded growth synthesis of citrate-stabilized gold nanoparticles of up to 200 nm: size focusing versus Ostwald ripening. *Langmuir* **2011,** *27* (17), 11098−11105.
2. Dong, J.-C.; Zhang, X.-G.; Briega-Martos, V.; Jin, X.; Yang, J.; Chen, S.; Yang, Z.-L.; Wu, D.-Y.; Feliu, J. M.; Williams, C. T.; Tian, Z.-Q.; Li, J.-F., In situ Raman spectroscopic evidence for oxygen reduction reaction intermediates at platinum single-crystal surfaces. *Nat. Energy* **2018,** *4* (1), 60−67.
3. Zhang, K.; Liu, Y.; Wang, Y.; Zhao, J.; Liu, B., Direct SERS tracking of a chemical reaction at a single 13 nm gold nanoparticle. *Chem. Sci.,* **2019,** *10* (6), 1741−1745.
4. Motl, N. E.; Ewusi-Annan, E.; Sines, I. T.; Jensen, L.; Schaak, R. E., Au−Cu Alloy Nanoparticles with Tunable Compositions and Plasmonic Properties: Experimental Determination of Composition and Correlation with Theory. *J. Phys. Chem. C* **2010,** *114* (45), 19256−19269.
5. Xie, W.; Walkenfort, B.; Schlucker, S., Label-free SERS monitoring of chemical reactions catalyzed by small gold nanoparticles using 3D plasmonic superstructures. *J. Am. Chem. Soc.* **2013,** *135* (5), 1657−1660.
6. Zhang, J.; Winget, S. A.; Wu, Y.; Su, D.; Sun, X.; Xie, Z. X.; Qin, D., Ag@Au Concave Cuboctahedra: A Unique Probe for Monitoring Au-Catalyzed Reduction and Oxidation Reactions by Surface-Enhanced Raman Spectroscopy. *ACS Nano* **2016**, *10 (2)*, 2607−2616.
7. Liu, J.; Liu, Z.; Wang, W.; Tian, Y., Real-time Tracking and Sensing of Cu^{+} and Cu^{2+} with a Single SERS Probe in the Live Brain: Toward Understanding Why Copper Ions Were Increased upon Ischemia. *Angew. Chem. Int. Ed.* **2021**, *60 (39)*, 21351−21359.
8. Kresse, G.; Furthmüller, J. Efficiency of Ab-Initio Total Energy Calculations for Metals and Semiconductors Using a Plane-Wave Basis Set. Comput. Mater. Sci. 1996, 6, 15−50.
9. Kresse, G.; Furthmüller, J. Efficient Iterative Schemes for Ab Initio Total-Energy Calculations Using a Plane-Wave Basis Set. Phys. Rev. B 1996, 54, 11169−11186.
10. Perdew, J. P.; Burke, K.; Ernzerhof, M. Generalized Gradient Approximation Made Simple. Phys. Rev. Lett. 1996, 77, 3865−3868.
11. Kresse, G.; Joubert, D. From Ultrasoft Pseudopotentials to the Projector Augmented-Wave Method. Phys. Rev. B 1999, 59, 1758-1775.
12. Blöchl, P. E. Projector Augmented-Wave Method. Phys. Rev. B 1994, 50, 17953−17979.
13. Wang, X.; Liu, G. K.; Xu, M. X.; Ren, B.; Tian Z. Q. Development of Weak Signal Recognition and an Extraction Algorithm for Raman Imaging. Anal. Chem. 2019, 91, 12909-12916.
